# How to approach multiple arrhythmias in a young athlete with *SCN5A* mutation

**DOI:** 10.1016/j.hrcr.2025.01.006

**Published:** 2025-04-15

**Authors:** Andreas Müssigbrodt, Romain Vergier, Astrid Monfort, Jocelyn Inamo, Gilles Millat, Patrice Bouvagnet

**Affiliations:** 1 Department of Cardiology, Centre Hospitalier Universitaire Martinique, Fort de France, France; 2 Unité Fonctionnelle Cardiogénétique, Moléculaire, Centre de Biologie et Pathologie Est, Hospices Civils de Lyon, Bron, France

**Keywords:** *SCN5A*, Brugada, Mutation, Atrial flutter, Conduction system, ICD, Pacemaker

Test your knowledge! Take an interactive quiz related to this article: https://www.heartrhythmcasereports.com/content/quiz_archive

## Introduction

Atrial fibrillation (AF) and atrial flutter (AFL) may be associated with a genetic predisposition, particularly in people younger than 45 years without obvious risk factors.^1^^,^^2^ In such cases, genetic testing may be considered to identify potential underlying genetic causes.^1^ It has been demonstrated that mutations in *SCN5A*, the gene encoding the α subunit of the human cardiac sodium channel (Na_v_1.5), may have pleiotropic effects, including risk of AF and AFL, but also Brugada syndrome (BrS), type 3 long QT syndrome, progressive cardiac conduction disease (PCCD), sick sinus syndrome (SSS), atrial standstill, sudden cardiac arrest (SCA), sudden infant death syndrome, and dilated and arrhythmogenic cardiomyopathy.^3^^,^^4^ Patients may be asymptomatic or present with symptoms such as palpitations, syncope, heart failure, and/or SCA.

We describe the clinical presentation and management of typical right AFL, paroxysmal AF, SSS, and PCCD in a young athlete with *SCN5A* mutation, highlighting the importance of genetic counseling and surveillance in genetic cardiac conditions.

## Case report

**Figure 1:**
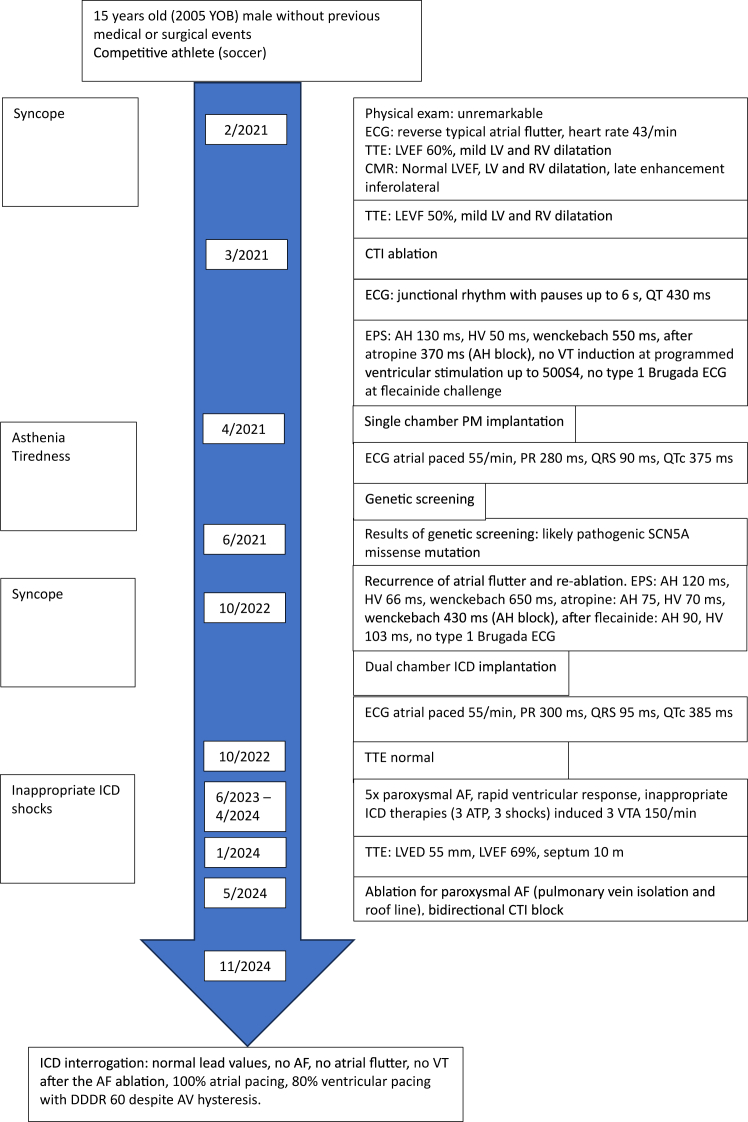
The timeline displays important disease-related events as symptoms, diagnostic measures, treatment, and follow-up of a young athlete with *SCN5A* mutation.

**Figure 2:**
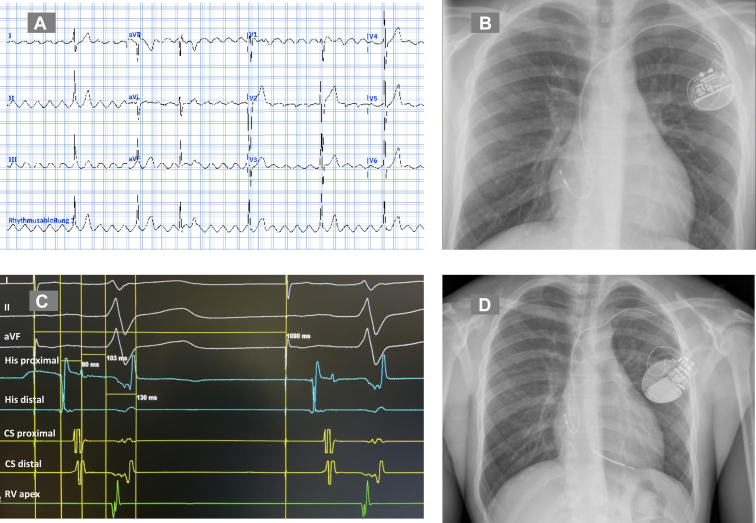
**A:** Clockwise typical atrial flutter in electrocardiogram (positive P waves in II, III, aVF, negative P wave in V1), positive Sokolow index as a sign of possible left ventricular hypertrophy, heart rate of 43 beats/min (sweep speed 25 mm/s). **B:** Single-chamber pacemaker implanted due to symptomatic sick sinus syndrome. **C:** Intracardiac electrograms during electrophysiological study. Paced cycle length of 1090 ms (55 beats/min), AH interval of 90 ms, HV interval of 103 ms, QRS of 130 ms after infusion of 120 mg flecainide. **D:** Dual-chamber ICD upgrade due to syncope, progressive cardiac conduction disease with HV progressing from 50 ms to 70 ms and HV >100 ms (after flecainide) and assumed risk of ventricular arrhythmias because of *SCN5A* mutation and late gadolinium enhancement in cardiac magnetic resonance tomography.

The young male patient was hospitalized at the age of 15 years after syncope occurring during soccer training (Figure 1). His physical examination was unremarkable. Electrocardiography (ECG) demonstrated clockwise typical AFL (Figure 2A). Transthoracic echocardiography demonstrated mild biventricular dilatation at normal left ventricular ejection fraction (LVEF). Cardiac magnetic resonance tomography confirmed the echocardiographic findings of LV and right ventricular dilatation at normal LVEF and revealed inferolateral late gadolinium enhancement.

He was scheduled for elective cavo-tricuspid isthmus ablation and discharged with 1.25 mg bisoprolol and 20 mg rivaroxaban (Figure 1).

One month later, ECG still demonstrated AFL. The athlete reported dyspnea at exercise. Transthoracic echocardiography displayed LVEF of 50% with mild dilatation of both ventricles.

Ablation of the cavo-tricuspid isthmus converted typical AFL into junctional escape rhythm at 30–40 beats/min, with evidence of bidirectional block. As conscious sedation, vagal activity or beta-blocker therapy were initially attributed to the junctional escape rhythm with bradycardia, bisoprolol was suspended (Figure 1).

Due to persistent bradycardia at 30–40 beats/min from slow junctional escape and sinus rhythms, with pauses lasting up to 6 seconds, an electrophysiological study (EPS) was performed 3 days later. Before the EPS, common modifiable causes for sinus node dysfunction were excluded. Thyroid function and potassium were normal, the patient did not take any bradycardia-causing drugs. At EPS, AH interval was 130 ms and HV interval was 50 ms. Wenckebach was measured at 550 ms before, and at 370 ms after, 2 mg of atropine. No supraventricular tachycardia could be induced. No ventricular tachyarrhythmia (VTA) could be induced with programmed ventricular stimulation from 2 different right ventricular pacing sites using 3 different drive cycle lengths with up to 3 extra-stimuli, with and without isoproterenol. There was no evidence of a Brugada type 1 ECG after infusion of 120 mg (2 mg/kg) of flecainide.

Lyme and Chagas serology were negative. At treadmill test, sinus rhythm accelerated until a maximal heart rate of 136 beats/min at 144 W. No QT prolongation with exercise or during recovery could be observed. Given the patient's symptoms of fatigue, which were attributed to SSS with chronotropic incompetence, we decided to implant a single-chamber pacemaker with an atrial lead (Figure 2B). ECG after implantation demonstrated atrial pacing at 55 beats/min, PR interval of 280 ms, QRS interval of 90 ms, and corrected QTc interval of 375 ms. Transthoracic echocardiography was normal, with LVEF of 70% (Figure 1).

Eighteen months later, symptomatic typical AFL recurred. Another cavo-tricuspid isthmus ablation was performed, with evidence of bidirectional block at the end of the procedure. EPS demonstrated AH interval of 120 ms, HV interval of 66 ms, and a Wenckebach of 650 ms with AH block before 2 mg atropine. After injection of 2 mg atropine, AH interval was at 75 ms, HV interval was at 70 ms, and a Wenckebach of 430 ms. After infusion of 120 mg (2 mg/kg) of flecainide, there was no evidence of Brugada type 1 ECG, AH interval was at 93 ms, HV interval 103 ms (Figure 2C), and Wenckebach at 430 ms with AH block.

Genetic sequencing of 46 arrhythmogenic genes (see Supplemental Data) revealed a previously undescribed heterozygous missense variant NC_000003.12:g.38560355A>T ; q:c.4034T>A ; NP_000326.2:p.(Leu1345His) of the *SCN5A* gene with likely pathogenic significance (Supplemental Data). This variant is absent from gnomAD and ClinVar databases of genetic variants. The amino acid is highly interspecies conserved up to *Xenopus tropicalis* (Supplemental Figure 1). At the same amino acid, another missense variant (p.Leu1345Pro) was classified as likely pathogenic in a case of BrS^5^ with supporting experimental data for pathogenicity^6^ (see Supplemental Data for variant nomenclature). Genetic screening was declined by the asymptomatic mother and sister of the patient. The family has no contact with the patient's father.

As the patient experienced another syncope after AAI pacemaker implantation and showed signs of PCCD in the EPS, we identified an indication for additional ventricular pacing. Given that syncope, SSS, PCCD, AF, and AFL are associated with VTA and SCA in young patients with SCN5A mutations,^3^^,^^7^ and considering the presence of late gadolinium enhancement on cardiac magnetic resonance tomography, we decided—together with the young patient and his family—to implant a dual-chamber implantable cardioverter-defibrillator (ICD) instead of a dual-chamber pacemaker (Figure 2D).

During 15 months of follow-up after ICD implantation, the patient experienced 5 paroxysmal AF episodes with rapid ventricular response and inappropriate ICD therapies during soccer training. Inappropriate shocks induced 3 episodes of VTA of 150 beats/min. An AF ablation with pulmonary vein isolation and roof line ablation were performed in another center. At 6 months follow-up after this ablation, no further AF, AFL, or VTA episodes were observed (Figure 1). The patient is doing well and regularly plays soccer during his leisure time.

## Discussion

### Anticoagulation in young individuals with AFL and AF and low thromboembolic risk score

Oral anticoagulation was prescribed for 4 weeks after restoration of sinus rhythm after AFL ablation and for 3 months after AF ablation, as proposed by current guidelines.^1^^,^^8^ It is unknown whether there is a benefit of a long-term anticoagulation in this young patient with *SCN5A* mutation. As the patient remained in sinus rhythm after the last ablation, and as atrial arrhythmias can be detected by telemonitoring of the dual-chamber ICD, we think that long-term anticoagulation is not indicated currently.

### Stratification and prevention of future arrhythmic events in individuals with SCN5A variants without Brugada ECG

Currently, there are no international guideline recommendations for primary prevention ICD indication for *SCN5A* variant carriers without Brugada phenotype. Risk stratification is complicated by the incomplete penetrance and variable expressivity of *SCN5A* channelopathy, attributed to both genetic and nongenetic factors.^9^ One prospective study of children with *SCN5A* variants included 442 neonates.^7^ Many children showed no ECG alterations at birth (44.3%) and the most frequent arrhythmogenic phenotype was isolated PCCD (25.6%), followed by overlap syndrome (15%), long QT syndrome (10.6%), and BrS (1.8%).^7^ Overlap syndrome involves a combination of 2 or more distinct clinical entities associated with SCN5A mutations, such as long QT syndrome, BrS, PCCD, and/or SSS.^7^ There were 272 cases of VTA, SCA, and/or syncope in 139 patients (31.5%).^7^ Overlap syndrome and PCCD, as seen in our case, increase the risk of SCA and VTA.^7^ SCA was the initial manifestation in 23.2% of 69 patients with overlap syndrome and 18% of 113 patients with isolated PCCD.^7^ More than one-half of patients with overlap syndrome who received an ICD (9 of 17 [53%]) experienced appropriate ICD shocks.^7^ Another study described 17 symptomatic, unrelated patients with *SCN5A* variants.^3^ Twelve of 17 patients demonstrated VTA.^3^ VTAs were associated with syncope, SSS, PCCD, AF, and AFL.^3^ Therefore, we propose that ICD prophylaxis should be considered for patients with pathogenic or likely pathogenic SCN5A variants and overlap syndrome or PCCD, even in the absence of VTA or reduced LVEF. In addition, late gadolinium enhancement on cardiac magnetic resonance tomography, as observed in our patient, is associated with adverse outcomes, including increased mortality and major cardiac events, independent of LVEF.^10^ This finding further justified our decision to proceed with ICD implantation.

### Return to play

Strenuous physical exercise during soccer training may have induced AF with rapid ventricular response, leading to inappropriate ICD shocks. In France, participation in competitive sports for individuals with ICDs is highly restricted, but can be authorized on a case-by-case basis. The European Society of Cardiology guidelines from 2020 and 2021, along with the Heart Rhythm Society expert consensus statement from 2024, provide well-balanced advice on managing this complex issue.^11^, ^12^, ^13^ After shared decision making, the patient chose to continue playing soccer recreationally, without engaging in competitive matches.

## Conclusion

The occurrence of AFL, AF, or significant bradyarrhythmia in young individuals before the age of 45 years and with no obvious risk factors should prompt genetic testing. This case of a young athlete with SSS, PCCD, AFL, and AF, but without Brugada ECG, underscores the heterogenous expression of *SCN5A* mutations. Evidence-based, individually adapted diagnostic and treatment, including arrhythmic risk assessment and genetic counseling, may help to improve outcomes and quality of life of patients with *SCN5A* mutations.

## Disclosures

The authors have no conflicts of interest to disclose.
