# Supplementary material for: How to approach multiple arrhythmias in a young athlete with SCN5A mutation
Source: HeartRhythm Case Rep. 2025 Apr 15;11(4):277–80. doi: 10.1016/j.hrcr.2025.01.006 (PMC12138029; doi:10.1016/j.hrcr.2025.01.006)
Supplement: Supplementary Data [file mmc1.docx]

**SUPPLEMENTARY DATA**

**Genes and transcripts which were sequenced** : *AKAP9* (NM_005751.4), *ANK2* (NM_001148.5), *CACNA1C* (NM_199460.3), *CACNA2D1* (NM_000722.3), *CACNB2* (NM_000724.3), *CALM1* (NM_006888.4), *CALM2* (NM_001743.6), *CALM3* (NM_005184.2), *CASQ2* (NM_001232.3), *CAV3* (NM_001234.4), *DES* (NM_001927.3), *DPP6* (NM_130797.3), *GJA1* (NM_000165.5), *GJA5* (NM_005266.6), *GPD1L* (NM_015141.3), *HCN4* (NM_005477.2), *KCNA5* (NM_002234.3), *KCND3* (NM_004980.4), *KCNE1* (NM_000219.5), *KCNE2* (NM_172201.1), *KCNE3* (NM_005472.4), *KCNE5* (NM_012282.3), *KCNH2* (NM_000238.3), *KCNJ2* (NM_000891.2), *KCNJ5* (NM_000890.4), *KCNJ8* (NM_004982.3), *KCNQ1* (NM_000218.2), *LMNA* (NM_170707.4), *NKX2-5* (NM_004387.3), *NOS1AP* (NM_014697.2), *NPPA* (NM_006172.3), *PRKAG2* (NM_016203.3), *RANGRF* (NM_016492.4), *RYR2* (NM_001035.2), *SCN10A* (NM_006514.3), *SCN1B* (NM_199037.4), *SCN2B* (NM_004588.4), *SCN3B* (NM_018400.3), *SCN4B* (NM_174934.3), *SCN5A* (NM_198056.2), *SLC4A3* (NM_201574.2), *SLMAP* (NM_007159.4), *SNTA1* (NM_003098.2), *TECRL* (NM_001010874.4), *TRDN* (NM_006073.3) and *TRPM4* (NM_017636.3).

The initial variant description of the patient reported in this article was: NM_198056.2(SCN5A) :c.4037T>A, p.(Leu1346His)

The up-dated variant description is:

- For NCBI : NC_000003.12:g.38560355A>T ; NM_000335.5:c.4034T>A ; NP_000326.2:p.(Leu1345His)
- For ENSEMBL : ENSG00000183873.19 ; ENST00000423572.7 : c.4034T>A ; ENSP00000398266.2 : p.(Leu1345His)

The initial variant description of the patient reported in articles (4) and (5) of the reference list is:

NM_198056.2(SCN5A) :c.4037T>C, p.(Leu1346Pro)

The up-dated description of the variant reported in articles (4) and (5) is:

- For NCBI : NC_000003.12:g.38560355A>G; NM_000335.5:c.4034T>C; NP_000326.2:p.(Leu1345Pro)
- For ENSEMBL : ENSG00000183873.19 ; ENST00000423572.7 : c.4034T>C ; ENSP00000398266.2 : p.(Leu1345Pro)

**gnomAD**: for genome Aggregation Database. The gnomAD database is composed of exome and genome sequences from around the world. Cohorts of pediatric disease were removed, except for a small number of diverse cohorts where only unaffected relatives were included. As such, the gnomAD resource should serve as useful reference sets of allele frequencies for severe pediatric disease studies - however, note that some individuals with severe disease may still be included in the data sets such as biobanks, albeit likely at a frequency equivalent to or lower than that seen in the general population.

**ClinVar** : ClinVar is a public archive with free access to reports on the relationships between human variations and phenotypes, with supporting evidence. The database includes germline and somatic variants of any size, type or genomic location

**ACMG (American College of Medical Genetics) classification** : Likely Pathogenic

- PM1: Located in a mutational hot spot and/or critical and well-established functional domain without benign variation

- PM2: Absent from controls

- PM5: Novel missense change at an amino acid residue where a different missense change determined to be pathogenic has been seen before

- PP2: missense variant in a gene that has a low rate of missense variation and in which missense variants are a common mechanism of disease

- PP3: Multiple computational lines of evidence support a deleterious effect on the gene or gene product (SIFT, REVEL, ClinPred, Meta SVM, Meta LR, Mistic: Damaging; PolyPhen2 HumDiv, PolyPhen2 HumVar: Probably damaging, AlphaMissense: Likely Pathogenic)

**Supplementary Figure 1** :


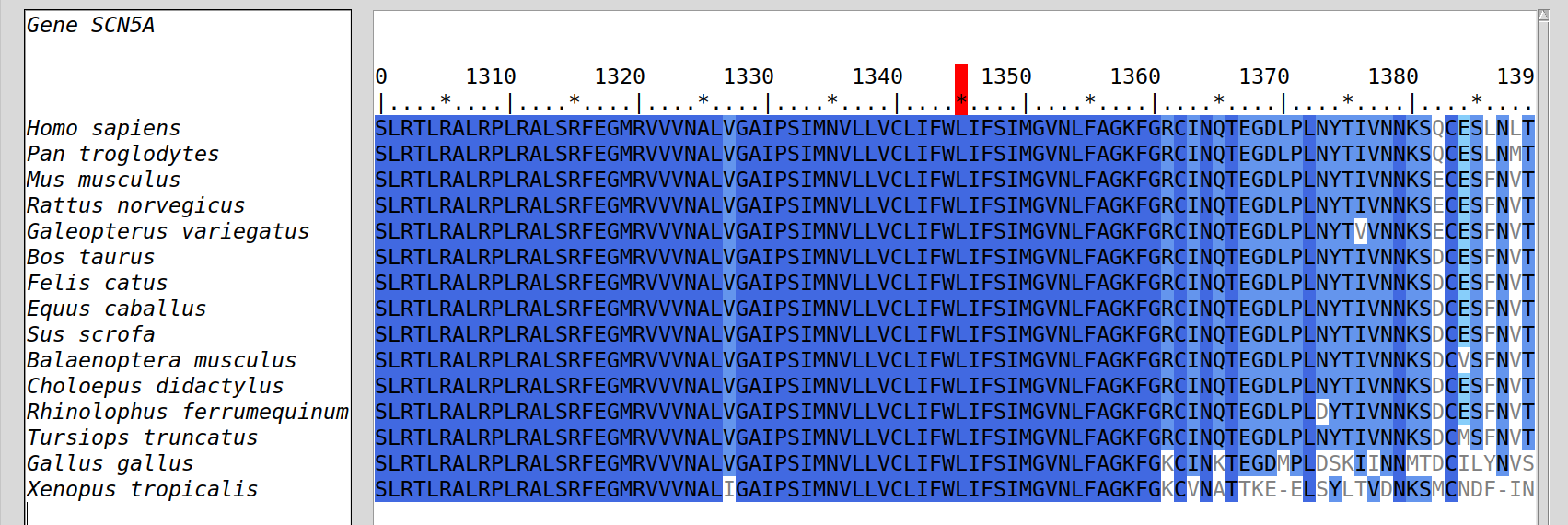


**Legend** : The peptide derived from the NM_000335.5 transcript of the SCN5A gene (NP_000326.2) was aligned to the orthologous peptide of 14 different species (*Pan troglodytes* (XP_016796233.2); *Mus musculus* (XP_006512058.1); *Rattus norvegicus* (NP_037257.2); *Galeopterus variegatus* (XP_008565387.1); *Bos taurus* (XP_024838162.1); *Felis catus* (A0A2I2UMY5); *Equus caballus* (XP_023475508.1); *Sus scrofa* (XP_020927335.1); *Balaenoptera musculus* (XP_036725735.1); *Choloepus didactylus* (XP_037703380.1); *Rhinolophus ferrumequinum* (XP_032988997.1); *Tursiops truncatus* (XP_033719598.1); *Gallus gallus* (XP_015136737.3) and *Xenopus tropicalis* (XP_031760488.1). The p.Leu1345 residue is shown by a red rectangle. It is conserved in all species explored until *Xenopus tropicalis*.

The sequence IPSIMNVLLVCLIFW**L**IFSIMGVNL (amino acid 1331-1355) is the S5 transmembrane region of domain III. The Leucine residue is bolded. The extracellular pore-forming region is located between region S5 and S6 of domain repeat III.
